# Supplementary material for: Gene expression signatures in blood from a West African sepsis cohort define host response phenotypes
Source: Nat Commun. 2024 May 30;15:4606. doi: 10.1038/s41467-024-48821-0 (PMC11139862; doi:10.1038/s41467-024-48821-0)
Supplement: Supplementary file 1 — Supplementary Information [file 41467_2024_48821_MOESM1_ESM.pdf]

Supplementary Information

for

Gene Expression Signatures in Blood from a West African Sepsis Cohort Define Host Response Phenotypes

Josh G. Chenoweth[1], Carlo Colantuoni[2], Deborah A. Striegel[1], Pavol Genzor[1], Joost Brandsma[1], Paul W. Blair[1,3], Subramaniam Krishnan[1], Elizabeth Chiyka[1], Mehran Fazli[1], Rittal Mehta[1], Michael Considine[4], Leslie Cope[4], Audrey C. Knight[2], Anissa Elayadi[1], Anne Fox[5], Ronna Hertzano[6], Andrew G. Letizia[5], Alex Owusu-Ofori[7,8], Isaac Boakye[9], Albert A. Aduboffour[7], Daniel Ansong[10,11], Eno Biney[12], George Oduro[12], Kevin L. Schully[13], Danielle V. Clark[1].

[1] Austere environments Consortium for Enhanced Sepsis Outcomes (ACESO), The Henry M. Jackson Foundation for the Advancement of Military Medicine, Inc., Bethesda, Maryland, USA.
[2]Department of Neurology, Johns Hopkins School of Medicine, Baltimore, MD, USA.
[3]Department of Pathology, Uniformed Services University, Bethesda, MD.
[4]Sidney Kimmel Comprehensive Cancer Center, Johns Hopkins University, Baltimore, MD.
[5]Naval Medical Research Unit EURAFCENT Ghana detachment, Accra, Ghana.
[6]Section on Omics and Translational Science of Hearing, Neurotology Branch, National Institute on Deafness and Other Communication Disorders, National Institutes of Health, Bethesda, MD, USA.
[7]Laboratory Services Directorate, Komfo Anokye Teaching Hospital (KATH), Kumasi, Ghana.
[8]Department of Clinical Microbiology, Kwame Nkrumah University of Science and Technology (KNUST), Ghana.
[9]Research and Development Unit, KATH, Kumasi, Ghana.
[10]Child Health Directorate, KATH, Kumasi, Ghana.
[11]Department of Child Health, KNUST, Kumasi, Ghana.
[12]Accident and Emergency Department, KATH, Kumasi, Ghana.
[13]Austere environments Consortium for Enhanced Sepsis Outcomes (ACESO), Biological Defense Research Directorate, Naval Medical Research Command-Frederick, Ft. Detrick, Maryland, USA.

These authors contributed equally: Josh G. Chenoweth and Carlo Colantuoni.

Correspondence and requests should be addressed to Josh G. Chenoweth [jchenoweth@aceso-sepsis.org]

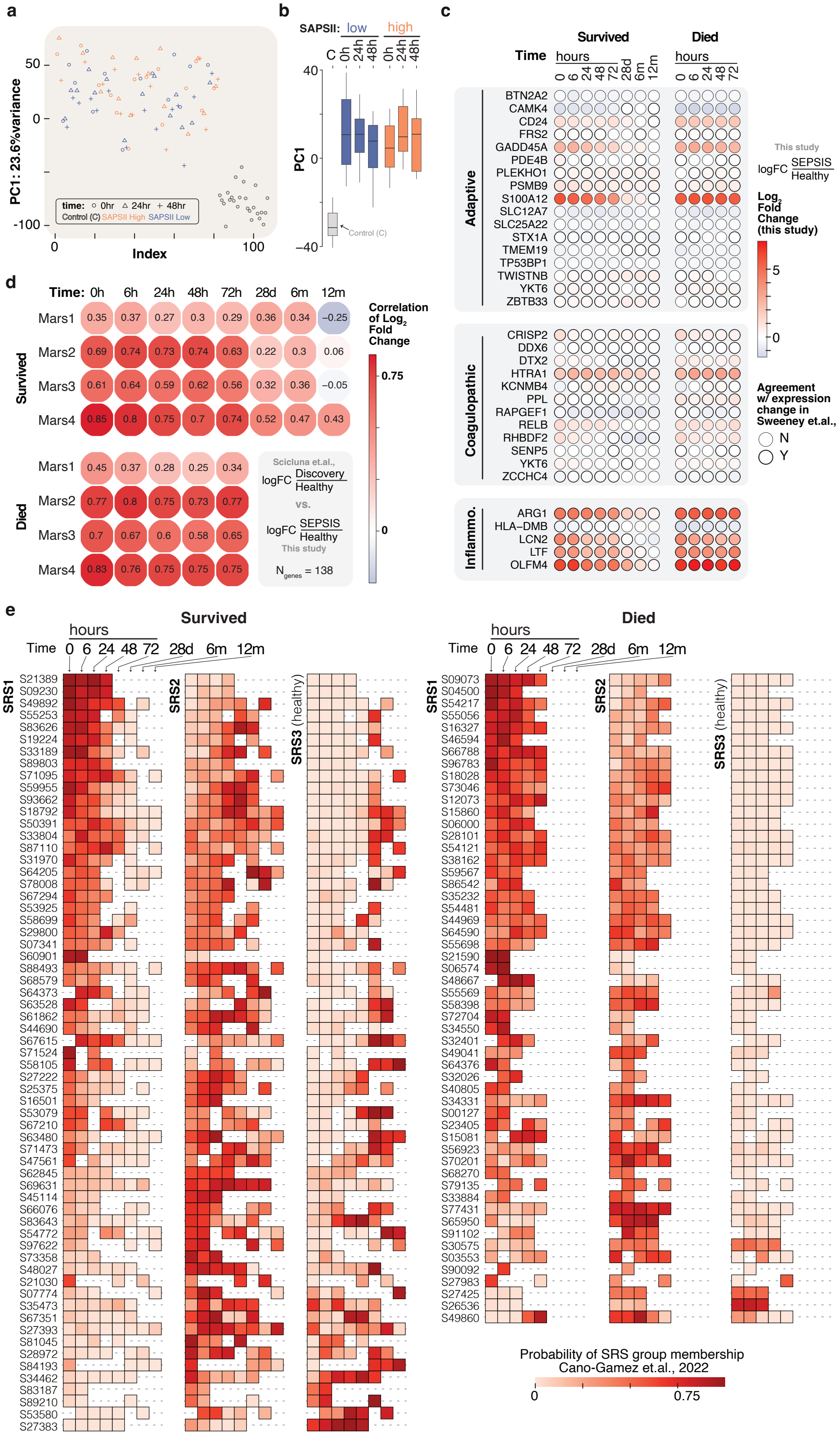
a
PC1: 23.6%variance
Index
50
0
-100
0
100
time: 0hr 24hr 48hr
Control (C) SAPSII High SAPSII Low
b
SAPSII: low high
C 0h 24h 48h 0h 24h 48h
PC1
40
0
−40
Control (C)
c
Survived
Died
Time
hours
0 6 24 48 72 28d 6m 12m
hours
0 6 24 48 72
Adaptive
BTN2A2
CAMK4
CD24
FRS2
GADD45A
PDE4B
PLEKHO1
PSMB9
S100A12
SLC12A7
SLC25A22
STX1A
TMEM19
TP53BP1
TWISTNB
YKT6
ZBTB33
Coagulopathic
CRISP2
DDX6
DTX2
HTRA1
KCNMB4
PPL
RAPGEF1
RELB
RHBDF2
SENP5
YKT6
ZCCHC4
Inflammo.
ARG1
HLA−DMB
LCN2
LTF
OLFM4
This study
logFC SEPSIS / Healthy
Log2 Fold Change (this study)
5
0
Agreement w/ expression change in Sweeney et.al.,
N
Y
d
Time: 0h 6h 24h 48h 72h 28d 6m 12m
Survived
Mars1 0.35 0.37 0.27 0.3 0.29 0.36 0.34 −0.25
Mars2 0.69 0.74 0.73 0.74 0.63 0.22 0.3 0.06
Mars3 0.61 0.64 0.59 0.62 0.56 0.32 0.36 −0.05
Mars4 0.85 0.8 0.75 0.7 0.74 0.52 0.47 0.43
Died
Mars1 0.45 0.37 0.28 0.25 0.34
Mars2 0.77 0.8 0.75 0.73 0.77
Mars3 0.7 0.67 0.6 0.58 0.65
Mars4 0.83 0.76 0.75 0.75 0.75
Scicluna et.al.,
logFC Discovery / Healthy
vs.
logFC SEPSIS / Healthy
This study
N genes = 138
Correlation of Log2 Fold Change
0.75
0
e
Survived
Died
hours
Time 0 6 24 48 72 28d 6m 12m
SRS1
SRS2
SRS3 (healthy)
S21389
S09230
S49892
S55253
S83626
S19224
S33189
S89803
S71095
S59955
S93662
S18792
S50391
S33804
S87110
S31970
S64205
S78008
S67294
S53925
S58699
S29800
S07341
S60901
S88493
S68579
S64373
S63528
S61862
S44690
S67615
S71524
S58105
S27222
S25375
S16501
S53079
S67210
S63480
S71473
S47561
S62845
S69631
S45114
S66076
S83643
S54772
S97622
S73358
S48027
S21030
S07774
S35473
S67351
S27393
S81045
S28972
S84193
S34462
S83187
S89210
S53580
S27383
S09073
S04500
S54217
S55056
S16327
S46594
S66788
S96783
S18028
S73046
S12073
S15860
S06000
S28101
S54121
S38162
S59567
S86542
S35232
S54481
S44969
S64590
S55698
S21590
S06574
S48667
S55569
S58398
S72704
S34550
S32401
S49041
S64376
S32026
S40805
S34331
S00127
S23405
S15081
S56923
S70201
S68270
S79135
S33884
S77431
S65950
S91102
S30575
S03553
S90092
S27983
S27425
S26536
S49860
Probability of SRS group membership
Cano-Gamez et.al., 2022
0
0.75

Supplementary Fig. 1. Public sepsis signatures and endotypes in the longitudinal Ghana sepsis cohort.

[a] PCA decomposition of the public sepsis data set (Cazalis et.al., 2014, GSE57065, PMID 26215705) which includes normal donors (Controls, grey circles), sepsis subjects with high SAPSII score (orange shapes), sepsis subjects with low SAPSII score (blue shapes). The collection time points include time 0h (circle), 24h (triangle), and 48h (cross). **[b]** Projection of the data set in [a] onto PC1 from the Ghanaian cohort in this study. **[c]** Expression of marker genes in the Ghanaian cohort (this study) corresponding to the Adaptive, Coagulopathic, and Inflammopathic sepsis subtypes identified in Sweeney et.al., (2018, PMID 295379885) study. Color fill corresponds to the expression change of sepsis versus healthy subjects, over time, for survivors and those that died by 28 days. The Sweeney et.al., genes that had the same directionality of expression change as this study are highlighted with thick circle borders. **[d]** The correlation comparing gene markers of MARS1 through 4 sepsis endotypes (138 of 140 genes used) described in Scicluna et.al., (2017, PMID 28864056) with genes in this study. The gene expression change (sepsis versus healthy) for survivors and those who died by 28 days over time was calculated, and Log2 Fold Changes were compared using Pearson correlation. **[e]** Every subject of this study was evaluated using the SepstratifieR machine learning algorithm (Cano-Gamez et.al., 2022, PMID 36322631). The probability of membership in one of the sepsis response groups (SRS1, SRS2, SRS3 – healthy) for each subject and each timepoint was plotted as a heatmap. The probability of survivors being in the SRS3 group increased over time, however, most of those who died by 28 days remained in either the SRS1 or SRS2 groups. Exact sample numbers (n) for figure panels are described in Supplementary Data 2. The boxplots in [b] describe the median (middle horizontal line), 1st and 3rd quartiles (bottom and top of box respectively), and data minimum and maximum (vertical whiskers).

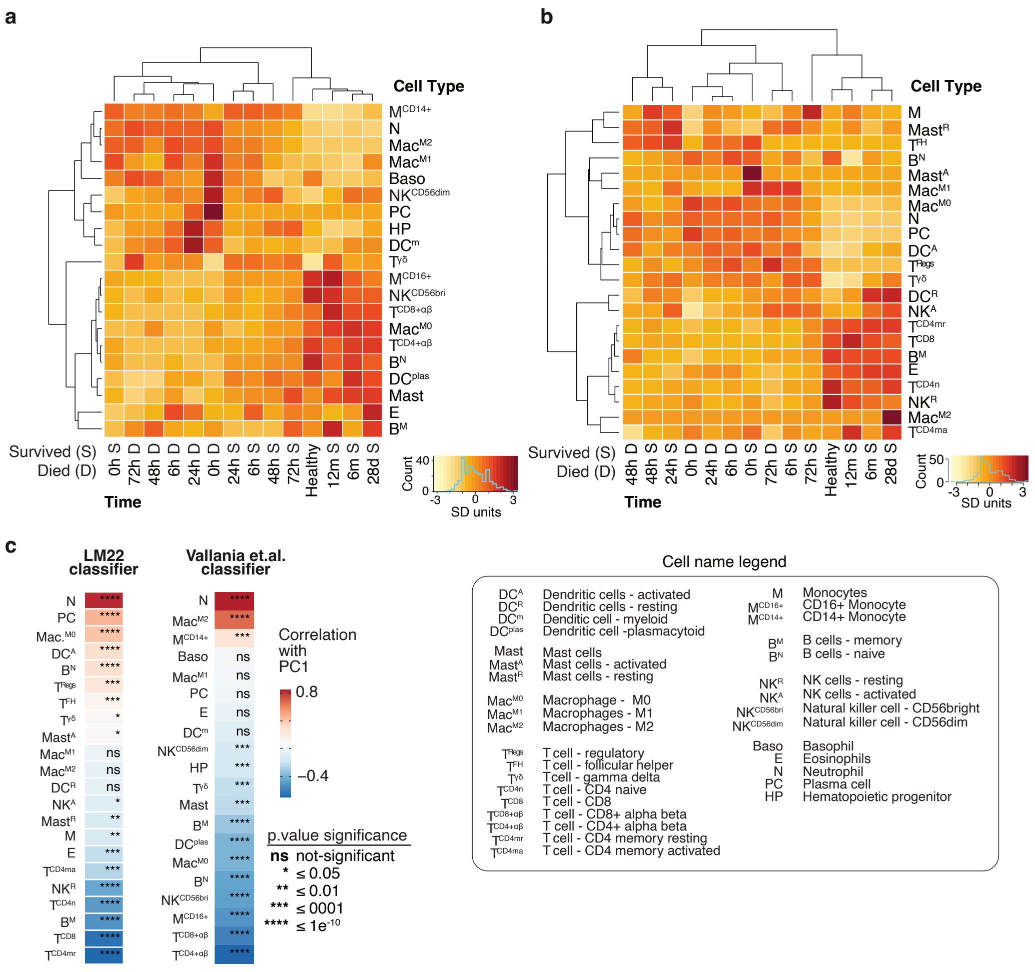

Supplementary Fig. 2. Deconvolution of bulk RNA-Seq data using CIBERSORT.
[a] Heatmap of average sample cell type estimates from CIBERSORT using the classifier from Vallania et.al., (2018, PMID 30413720). The columns and rows were clustered using unsupervised hierarchical clustering with a distance equal to 1 – Pearson correlation. **[b]** Same as in [a] but using classifier from Newman et.al., (2015, PMID 25822800) **[c]** Pearson correlation of sample PC1 scores with the cell type classifiers used in CIBERSORT deconvolution [a-b].Sample numbers (n) and statistical values for figure panels are described in Supplementary Data 2

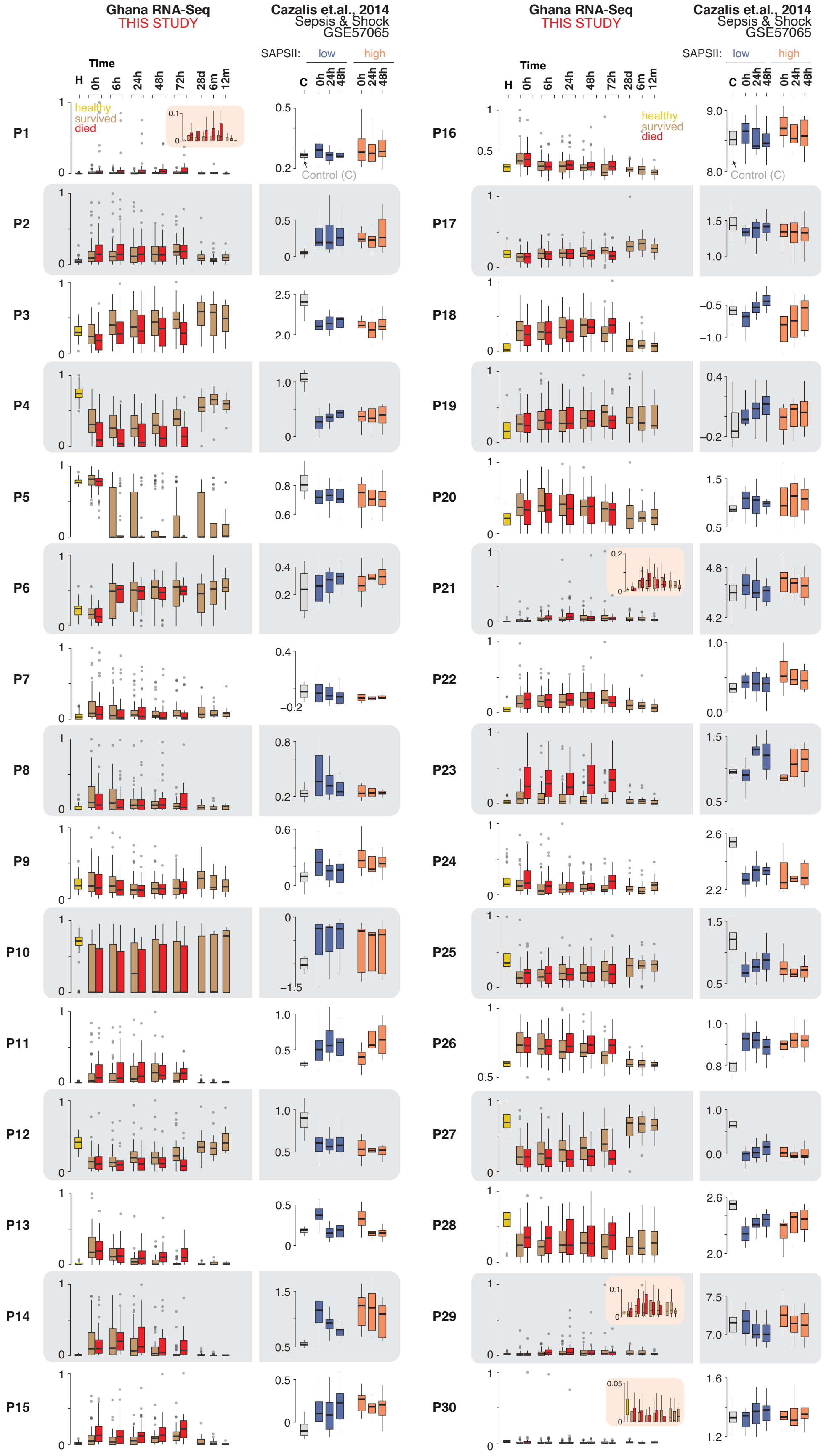

Ghana RNA-Seq
THIS STUDY
Cazalis et.al., 2014
Sepsis & Shock
GSE57065
SAPSII: low high
Time
H 0h 6h 24h 48h 72h 28d 6m 12m
C 0h 24h 48h 0h 24h 48h
healthy
survived
died
Control (C)
P1 P2 P3 P4 P5 P6 P7 P8 P9 P10 P11 P12 P13 P14 P15
P16 P17 P18 P19 P20 P21 P22 P23 P24 P25 P26 P27 P28 P29 P30

Supplementary Fig. 3. Individual CoGAPS gene expression patterns through time and public data projection.

The CoGAPS pattern values (left columns) were plotted as boxplots divided into groups as healthy (gold), surviving (tan), and subjects who died by 28 days (red) over time (0, 6, 24, 48, 72, 28 days, 6 and 12 months). The Cazalis et.al., (2014, GSE57065, PMID 26215705, right columns) data was projected onto CoGAPS pattern data. Normal donors (Controls, grey), sepsis subjects with high SAPSII scores (orange), and sepsis subjects with low SAPSII scores (blue). Sample numbers (n) and statistical values for figure panels are described in Supplementary Data 2. All boxplots describe the median (middle horizontal line), 1st and 3rd quartiles (bottom and top of box respectively), and data minimum and maximum (vertical whiskers).

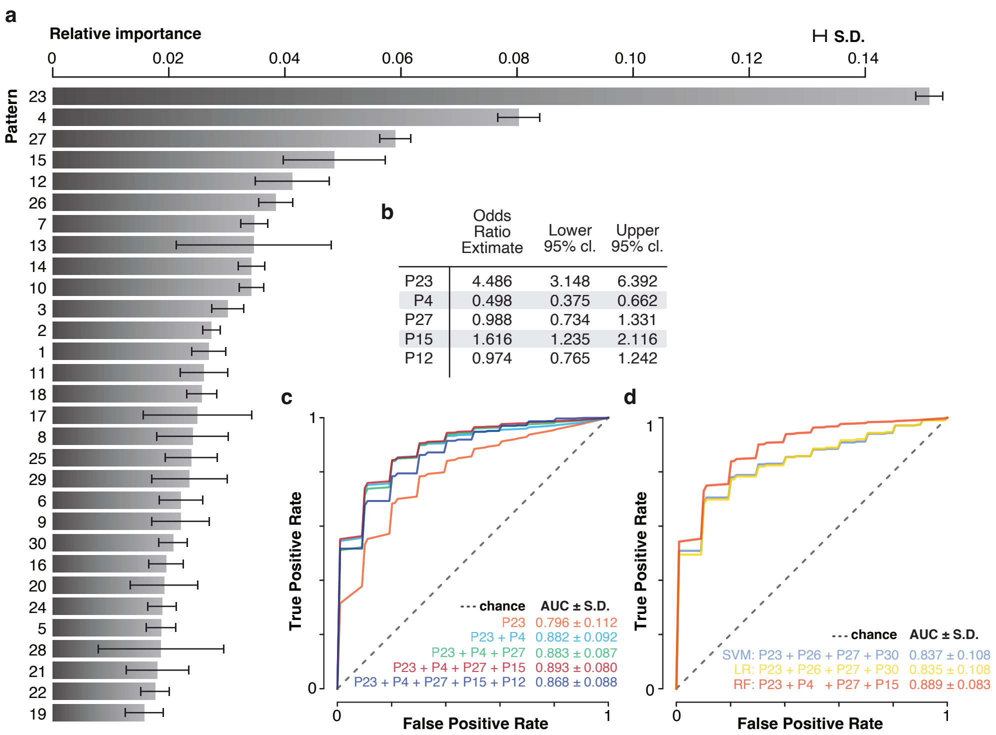

	Odds Ratio Extimate	Lower 95% cl.	Upper 95% cl.
P23	4.486	3.148	6.392
P4	0.498	0.375	0.662
P27	0.988	0.734	1.331
P15	1.616	1.235	2.116
P12	0.974	0.765	1.242

Supplementary Fig. 4. CoGAPS patterns confer risk or protection for sepsis 28-day mortality.

[a] Random forest model using 70/30 split for the training and testing sets, as well as stratified k-fold cross-validation with 10 folds and 10 repeats, implemented across all patterns. Variable importance was measured in the training set via Gini importance. Error bar corresponds to one standard deviation (S.D.) **[b]** Table showing results of analysis for pattern protection or risk using odds ratios method. Shown are 95% confidence intervals generated using logistic regression on the most important patterns. **[c]** Performance of the most important CoGAPS patterns in the testing set was measured via the area under the receiver operating characteristic curve (AUROC). Area under the curve (AUC) with one standard deviation (S.D.) is shown. **[d]** Comparison of performance of four most important patterns via AUROC analysis using three different modeling methods (SVM: support vector machine, LR: logistic regression; RF: random forest). Random forest achieives best performance (0.887 +/- 0.087) in this comparison. Sample numbers (n), statistical values, and source data for figure panels are described in Supplementary Data 2.

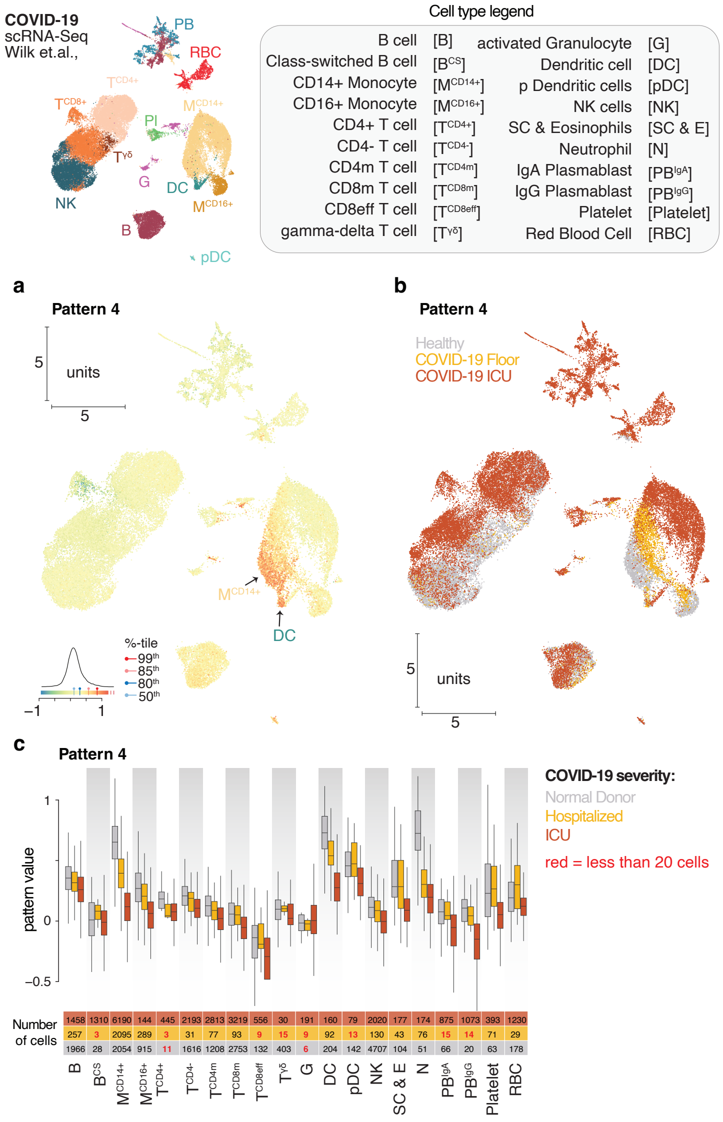

Supplementary Fig. 5. CoGAPS pattern 4 is depleted in myeloid cells of severely ill COVID-19 subjects.

[a] Projection of single-cell RNA-Seq data describing immune cells in COVID-19 (Wilk et.al., 2020, PMID 32514174) onto pattern 4. UMAP projections are colored by the magnitude of the CoGAPS pattern values in individual cells. The color gradient bar shows the range of projected values, percentile pins (%-tile) show selected percentile cutoffs, and the histogram depicts the distribution of all pattern values. The primary enriched cell types for pattern 4 are dendritic cells and CD14+ Monocytes (arrows and labels). **[b]** Projection of COVID-19 severity data from Wilk et.al., 2020 onto pattern 4. The colors indicate the cells from either healthy (grey), hospitalized (orange), or ICU (dark red) patients. **[c]** Boxplot of Wilk et.al., data (2020) showing the number of cells of each cell type in pattern 4. Color fill marks the normal donors (grey), hospitalized patients (orange), and ICU patients (dark red). Red number labels indicate cases with less than 20 cells. Sample numbers (n) and statistical values for figure panels are described in Supplementary Data 2. All boxplots describe the median (middle horizontal line), 1st and 3rd quartiles (bottom and top of box respectively), and data minimum and maximum (vertical whiskers).

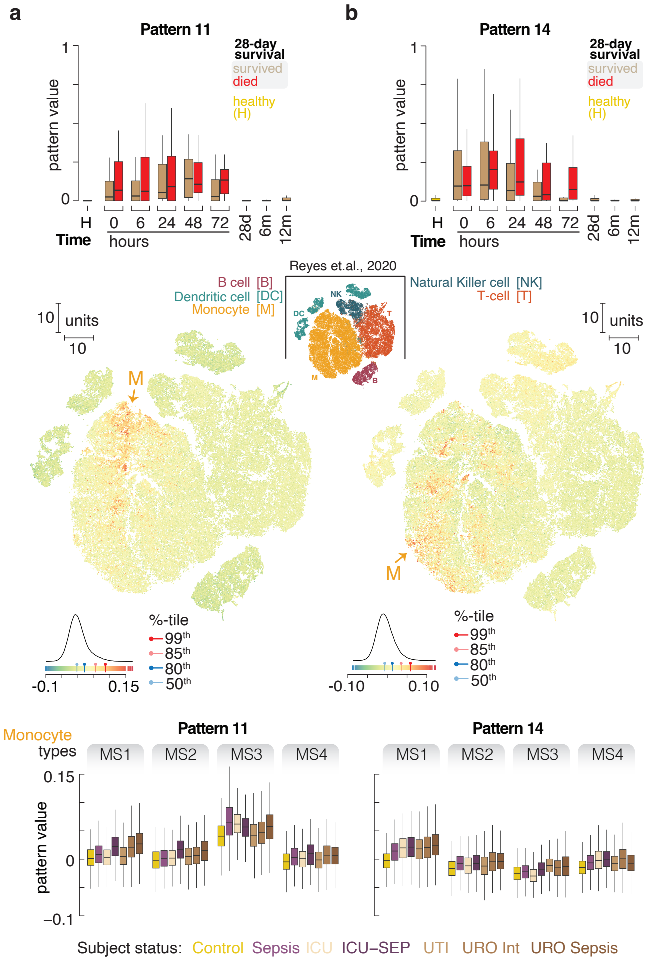

Supplementary Fig. 6. Patterns 11 and 14 show monocyte immunophenotypes.
[a] Boxplot of CoGAPS pattern 11 which is depleted in healthy donors (gold fill) as compared to survivors (tan), and those who died by 28 days (red). The middle panel shows a projection of scRNA-Seq sepsis data published by Reyes et.al., 2020 (PMID 32066974) onto pattern 11. The color gradient bar shows the range of projected values, percentile pins (%-tile) show selected percentile cutoffs, and the histogram depicts the distribution of all pattern values. A subset of monocytes is highlighted with an arrow and label (M). The boxplots on the bottom show projection of different monocyte populations in Reyes et.al., grouped by subject status into Controls (gold), Sepsis=hospitalized bacteremia (light purple), ICU=Intensive care (light tan), ICU-Sepsis=intensive care plus sepsis (dark purple), UTI=Urinary tract infection in the emergency department (light brown), Int-Uro=UTI with mild or transient organ dysfunction (brown), Uro-sepsis=UTI with clear or persistent organ dysfunction (dark brown) onto pattern 11. The most prominent group of monocytes is MS3. **[b]** Same analysis as in [a] but for pattern 14. The predominant type of monocytes is MS1. Sample numbers (n) and statistical values for figure panels are described in Supplementary Data 2. All boxplots describe the median (middle horizontal line), 1st and 3rd quartiles (bottom and top of box respectively), and data minimum and maximum (vertical whiskers).

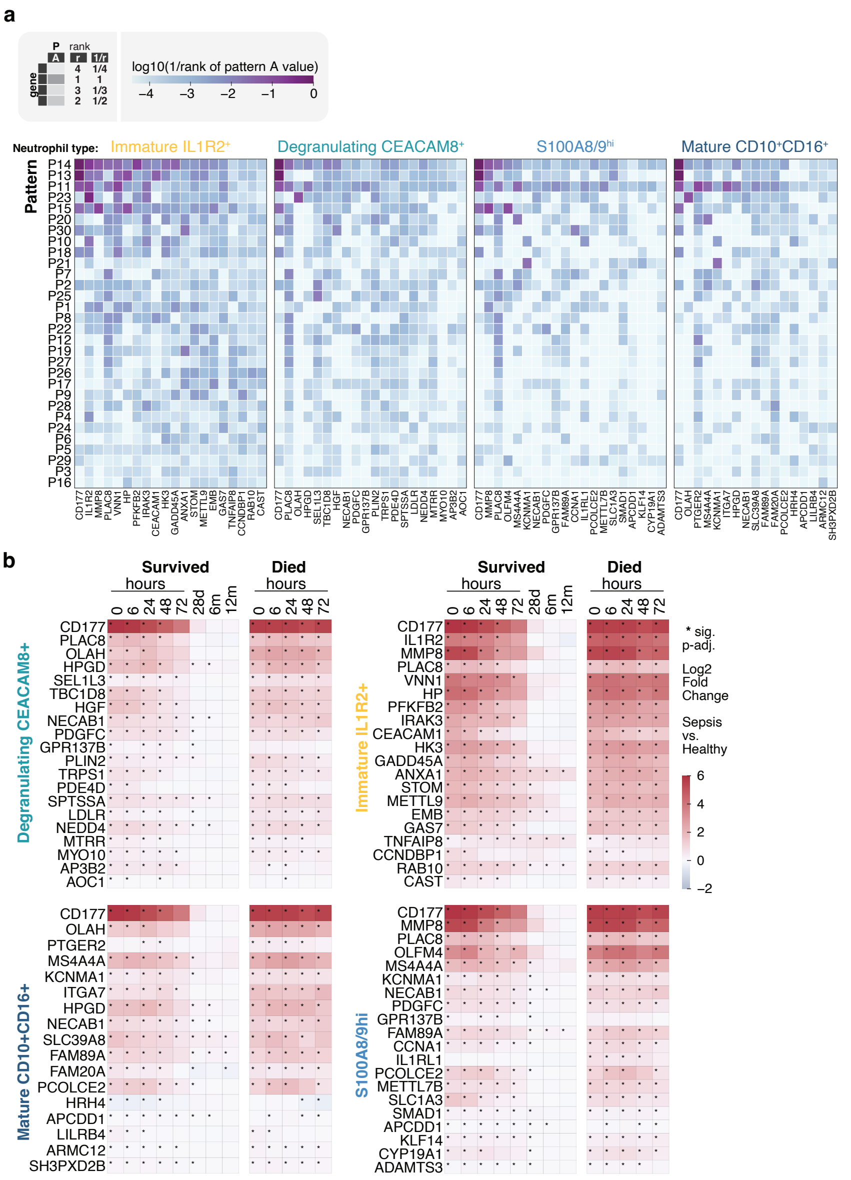

Supplementary Fig. 7. Neutrophil subtypes in CoGAPS patterns

[a] Heatmaps showing the ranks of the top neutrophil subtype genes from Kwok et.al 2023 (PMID 37095375) in each pattern. The top 20 genes enriched in sepsis in Kwok et.al., were used to identify the neutrophil subtypes except for mature neutrophils where only 17 genes matched. The top 5 patterns where neutrophil subtype genes are highly ranked are P14, P13, P11, P23, and P15 (as indicated by the intensity of the fill). **[b]** Expression changes of genes linked to different neutrophil populations were shown to be enriched in sepsis by Kwok et.al., 2023 in Ghana sepsis cohort (this study). Color fill corresponds to log2 fold changes of sepsis versus healthy subjects for survivors and those who died at 28 days. Significant adjusted p-values (two-sided Student's t-test, p-adjusted <0.05) are indicated by an asterisk (*). Sample numbers (n) and statistical values for figure panels are described in Supplementary Data 2. All boxplots describe the median (middle horizontal line), 1st and 3rd quartiles (bottom and top of box respectively), and data minimum and maximum (vertical whiskers).
