# Supplementary material for: Gene expression signatures in blood from a West African sepsis cohort define host response phenotypes
Source: Nat Commun. 2024 May 30;15:4606. doi: 10.1038/s41467-024-48821-0 (PMC11139862; doi:10.1038/s41467-024-48821-0)
Supplement: Supplementary file 3 — Description of Additional Supplementary Files [file 41467_2024_48821_MOESM3_ESM.pdf]

Description of Additional Supplementary Files

**Supplementary Data 1**	Summary and statistics related to the Healthy and Sepsis cohorts used throughout the study. Sheet 1 - entire cohort compared by gender. Sheet 2 - sepsis cohort with known 28-day mortality compared by 28-day mortality.
**Supplementary Data 2**	Sheet 1 - summary of the sample counts used in different analyses in manuscript. Sheet 2 - information about the source data for each figure.
