# Supplementary material for: Gene expression signatures in blood from a West African sepsis cohort define host response phenotypes
Source: Nat Commun. 2024 May 30;15:4606. doi: 10.1038/s41467-024-48821-0 (PMC11139862; doi:10.1038/s41467-024-48821-0)
Supplement: Supplementary file 6 — Reporting Summary [file 41467_2024_48821_MOESM6_ESM.pdf]

nature portfolio

Corresponding author(s): Josh G. Chenoweth

Last updated by author(s): 04/22/2024

Reporting Summary

Nature Portfolio wishes to improve the reproducibility of the work that we publish. This form provides structure for consistency and transparency in reporting. For further information on Nature Portfolio policies, see our Editorial Policies and the Editorial Policy Checklist.

Statistics

For all statistical analyses, confirm that the following items are present in the figure legend, table legend, main text, or Methods section.

n/a	Confirmed	
☐	☒	The exact sample size (n) for each experimental group/condition, given as a discrete number and unit of measurement
☐	☒	A statement on whether measurements were taken from distinct samples or whether the same sample was measured repeatedly
☐	☒	The statistical test(s) used AND whether they are one- or two-sided<br>*Only common tests should be described solely by name; describe more complex techniques in the Methods section.*
☐	☒	A description of all covariates tested
☒	☐	A description of any assumptions or corrections, such as tests of normality and adjustment for multiple comparisons
☐	☒	A full description of the statistical parameters including central tendency (e.g. means) or other basic estimates (e.g. regression coefficient) AND variation (e.g. standard deviation) or associated estimates of uncertainty (e.g. confidence intervals)
☐	☒	For null hypothesis testing, the test statistic (e.g. F, t, r) with confidence intervals, effect sizes, degrees of freedom and P value noted<br>*Give P values as exact values whenever suitable.*
☐	☒	For Bayesian analysis, information on the choice of priors and Markov chain Monte Carlo settings
☒	☐	For hierarchical and complex designs, identification of the appropriate level for tests and full reporting of outcomes
☐	☒	Estimates of effect sizes (e.g. Cohen's d, Pearson's r), indicating how they were calculated

Our web collection on statistics for biologists contains articles on many of the points above.

Software and code

Policy information about availability of computer code

Data collection

Protein results were collected using:

Discovery Workbench 4.0.12
Softmax Pro 7 GxP

No other data collection software was used.
No custom code has been developed for data collection or analysis.

Data analysis

Raw sequence data and processed subject gene level data used in this study have been deidentified and deposited in dbGaP (Accession: phs003608.v1.p1) under restricted access in compliance with study informed consent and National Institutes of Health Human Subjects Protection guidelines.

The processed deidentified data used to perform analyses and generate figures throughout the study can be accessed in Zenodo (accession: https://doi.org/10.5281/zenodo.10916993).

Limited computational methods are available at GitHub (https://github.com/HJF-ACESO/Sepsis_Ghana/).

Data visualization (https://nemoanalytics.org/p?l=ChenowethEtAl2024&g=CEBPA), and latent space exploration (https://nemoanalytics.org/p?p=p&l=ChenowethEtAl2024&c=GhanaSepsisCoGAPSp30&algo=nmf) can be accessed through the Neuroscience Multi-Omic Analytics (NEMO) Portal.

Data analysis was carried out using following computational packages:

Raw seq. processing, genome alignment, and gene quantification:
bcl2fastq version 2.17
Human Genome version hg38
STAR version 2.7.5a
RSEM version 1.3.3

R packages:
data.table v1.14.10
projectR v1.16.0
CoGAPS v3.21.3
limma v3.56.2
ggplot2 v3.4.4
ggrastr v1.0.2
SepstratifieR v1.0
table1 v1.4.3

Python packages:
sklearn v1.3.0
statsmodels v0.13.2
numpy v1.21.5
matplotlib v3.5.1

For manuscripts utilizing custom algorithms or software that are central to the research but not yet described in published literature, software must be made available to editors and reviewers. We strongly encourage code deposition in a community repository (e.g. GitHub). See the Nature Portfolio guidelines for submitting code & software for further information.

Data

Policy information about availability of data

All manuscripts must include a data availability statement. This statement should provide the following information, where applicable:

- Accession codes, unique identifiers, or web links for publicly available datasets
- A description of any restrictions on data availability
- For clinical datasets or third party data, please ensure that the statement adheres to our policy

Raw sequence data and processed subject gene level data used in this study have been deidentified and deposited in dbGaP (Accession: phs003608.v1.p1) under restricted access in compliance with study informed consent and National Institutes of Health Human Subjects Protection guidelines. Data can be obtained following local IRB approval and with a letter of collaboration with the primary study investigator(s).

The processed deidentified data used to perform analyses and generate figures throughout the study can be accessed in Zenodo (accession: https://doi.org/10.5281/zenodo.10916993). A description of available processed and source data is provided in the Supplementary Information with this manuscript.

Limited computational methods are available at GitHub (https://github.com/HJF-ACESO/Sepsis_Ghana/). Any additional scripts used for data analysis and plotting will be provided upon reasonable request to the corresponding author.

Data visualization (https://nemoanalytics.org/p?l=ChenowethEtAl2024&g=CEBPA), and latent space exploration (https://nemoanalytics.org/p?p=p&l=ChenowethEtAl2024&c=GhanaSepsisCoGAPSp30&algo=nmf) can be accessed through the Neuroscience Multi-Omic Analytics (NEMO) Portal.

Public data set accessions used in analyses.
Cazalis et.al., 2014 - GSE57065
Wilk et.al., 2020 - GSE150728
Reyes et.al., 2024 via single-cell portal at Broad Institute - SCP548

All other public data used in the analyses was accessed via publication links described in references.

Research involving human participants, their data, or biological material

Policy information about studies with human participants or human data. See also policy information about sex, gender (identity/presentation), and sexual orientation and race, ethnicity and racism.

Reporting on sex and gender	Self-reported sex information was collected as a part of the study protocol described previously in Blair PW, Mehta R, Oppong CK, et al. Screening tools for predicting mortality of adults with suspected sepsis: an international sepsis cohort validation study BMJ Open 2023;13:e067840. doi: 10.1136/bmjopen-2022-067840<br><br>Sex information was not considered on individual basis or to draw major conclusions.
Reporting on race, ethnicity, or other socially relevant groupings	No categorization variables were used.<br><br>Various study population characteristics were collected as a part of the study protocol described previously in Blair PW, Mehta R, Oppong CK, et al Screening tools for predicting mortality of adults with suspected sepsis: an international sepsis cohort validation study BMJ Open 2023;13:e067840. doi: 10.1136/bmjopen-2022-067840

Population characteristics	Population characteristics (i.e. age or sex) were not considered in any of the analyses.
Recruitment	The Ghana cohort in this study has been previously described in Blair PW, Mehta R, Oppong CK, et al Screening tools for predicting mortality of adults with suspected sepsis: an international sepsis cohort validation study BMJ Open 2023;13:e067840. doi: 10.1136/bmjopen-2022-067840
Ethics oversight	Study protocol NMRC.2016.0004-GHA was approved by the Naval Medical Research Command (NMRC) Institutional Review Board in compliance with all applicable Federal regulations governing the protection of human subjects as well as host country IRBs. The protocol was approved by the Committee on Human Research, Publication, and Ethics (CHRPE) at Kwame Nkrumah University of Science & Technology. All procedures were in accordance with the ethical standards of the Helsinki Declaration of the World Medical Association. All patients, or their legally authorized representatives, provided written informed consent. Compensation was only provided to cover subject transportation costs for follow-up visits.

Note that full information on the approval of the study protocol must also be provided in the manuscript.

Field-specific reporting

Please select the one below that is the best fit for your research. If you are not sure, read the appropriate sections before making your selection.

☒ Life sciences ☐ Behavioural & social sciences ☐ Ecological, evolutionary & environmental sciences

For a reference copy of the document with all sections, see nature.com/documents/nr-reporting-summary-flat.pdf

Life sciences study design

All studies must disclose on these points even when the disclosure is negative.

Sample size	All available subjects in the Ghana cohort reported in Blair et. al were considered. This study was exploratory so final inclusion was 120 patients selected out of the previously reported 187 using a nested case-control design based on 28-day mortality outcome considering age and sex. Other criteria included the availability of longitudinal biospecimens and RNA quality following specimen extraction as well as cost constraints for RNA sequencing.
Data exclusions	From the 120 subjects included in this study, 3 were excluded from a majority of the analysis due to unknown mortality data which was a primary outcome.
Replication	No technical replicate RNA-Sequencing experiments were carried out due to limited amounts of the collected blood and to not cause undue burden to study participants. However we analyzed subjects longitudinally through time.
Randomization	Randomization was used in analyses that utilized cross-validation approaches and the 28-day mortality outcome was the only criteria used to maintain overall population distribution within the folds.
Blinding	Blinding strategy was not applicable to this study because of its observational and descriptive design.

Reporting for specific materials, systems and methods

We require information from authors about some types of materials, experimental systems and methods used in many studies. Here, indicate whether each material, system or method listed is relevant to your study. If you are not sure if a list item applies to your research, read the appropriate section before selecting a response.

Materials & experimental systems

n/a	Involved in the study	
☒	☐	Antibodies
☒	☐	Eukaryotic cell lines
☒	☐	Palaeontology and archaeology
☒	☐	Animals and other organisms
☒	☐	Clinical data
☒	☐	Dual use research of concern
☒	☐	Plants

Methods

n/a	Involved in the study	
☒	☐	ChIP-seq
☒	☐	Flow cytometry
☒	☐	MRI-based neuroimaging
